# Molecular characterization of* NDM-1*-producing *Pseudomonas aeruginosa* isolates from hospitalized patients in Iran

**DOI:** 10.1186/s12941-021-00482-3

**Published:** 2021-11-03

**Authors:** Mojtaba Shahin, Ali Ahmadi

**Affiliations:** 1 Molecular Biology Research Center, Systems Biology and Poisonings Institute, Baqiyatallah University of Medical Sciences, Tehran, Iran; 2 Present Address: Department of Medical Laboratory Sciences, Faculty of Medical Sciences, Arak Branch, Islamic Azad University, Arak, Iran

**Keywords:** Pseudomonas aeruginosa, *bla*_NDM-1_, DLST, MHT, MBL

## Abstract

**Background:**

The emergence of carbapenem-resistant *Pseudomonas aeruginosa* is one of the most important challenges in a healthcare setting. The aim of this study is double-locus sequence typing (DLST) typing of *bla*_NDM-1_ positive *P. aeruginosa* isolates.

**Methods:**

Twenty-nine *bla*_NDM-1_ positive isolates were collected during three years of study from different cities in Iran. Modified hodge test (MHT), double-disk synergy test (DDST) and double-disk potentiation test (DDPT) was performed for detection of carbapenemase and metallo-beta-lactamase (MBL) producing *bla*_NDM-1_ positive *P. aeruginosa* isolates. The antibiotic resistance genes were considered by PCR method. Clonal relationship of *bla*_NDM-1_ positive was also characterized using DLST method.

**Results:**

Antibiotic susceptibility pattern showed that all isolates were resistant to imipenem and ertapenem. DDST and DDPT revealed that 15/29 (51.8%) and 26 (89.7%) of *bla*_NDM-1_ positive isolates were MBL producing isolates, respectively. The presence of *bla*_OXA-10,_ *bla*_VIM*-2*_, *bla*_IMP-1_ and *bla*_SPM_ genes were detected in 86.2%, 41.4%, 34.5% and 3.5% isolates, respectively. DLST typing results revealed the main cluster were DLST 25-11 with 13 infected or colonized patients.

**Conclusions:**

The presence of *bla*_NDM-1_ gene with other MBLs encoding genes in *P. aeruginosa* is a potential challenge in the treatment of microorganism infections. DLST showed partial diversity among 29 *bla*_NDM-1_ positive isolates.

## Background

*Pseudomonas aeruginosa* is one of the most important hospital-acquired pathogens that causes miscellaneous opportunistic infections [1]. The emergence of multidrug-resistant (MDR: was defined as acquired non-susceptibility to at least one agent in three or more antimicrobial categories) and extremely drug resistant (XDR: was defined as non-susceptibility to at least one agent in all but two or fewer antimicrobial categories) *P. aeruginosa* isolates has been considered as a major concern for the treatment of infections caused by these isolates [2]. Carbapenemases are a wide spectrum group of beta-lactamase which hydrolyzes carbapenems to other b-lactams including monobactams, penicillins, and cephalosporins. Although carbapenems are a commonly last resort treatment used for MDR *P. aeruginosa* infection, the emergence of carbapenem-resistant *P. aeruginosa* is becoming a main public health concern and is associated with high rates of mortality and morbidity among hospitalized patients [3, 4].

Resistance to carbapenems can be related to producing carbapenemase enzymes such as serine carbapenemases and the MBLs encoding genes such as IMP, VIM, and NDM enzymes [5]. The MBLs encoding genes such as *bla*_VIM_ and *bla*_IMP_ are one of the most clinically important classes of beta-lactamases; but, discovered transmissible New Delhi metallo*-*beta*-*lactamase*-*1 (*NDM-1*) is becoming the most threatening carbapenemase, recently [6–8]. The *bla*_NDM-1_ producing strains are resistant to a wide-ranging of other antibiotic groups and transport numerous additional resistance genes such as genes encodings resistance to fluoroquinolones, aminoglycosides, sulfonamides, and macrolides. Furthermore, the *NDM‑1* enzyme is surfacing, resulting in almost whole resistance to antibiotics [8–10].

Molecular typing of *P. aeruginosa* is important to understand the local epidemiology, but it remains a challenging issue. The epidemiology of *P. aeruginosa* has been analyzed by an array of different typing methods such as Pulsed-field gel electrophoresis (PFGE) and Multilocus sequence typing (MLST) that are costly, required speci*fi*c technical abilities and time to consume [11]. The newly described double-locus sequence typing (DLST) methods based on the partial sequencing of two highly variable loci to typing *P. aeruginosa* isolates which allowed us to obtain an unambiguous and standardized definition of types [12]. DLST has remarkable discriminatory power, reproducibility and is able to recognize high-risk epidemic clones [12]. Although *bla*_NDM-1_ positive isolates are rare, knowledge of its occurrence is considered as a serious menace, however, this study is the first report of DLST typing of *bla*_NDM-1_ positive *P. aeruginosa* isolates obtained from different part of Iran.

## Methods

### Study design, sampling, and bacterial isolates

A cross-sectional study was conducted at three major teaching Hospitals (Ahvaz, Tehran, and Isfahan) in Iran during three-year period. In total, 369 non-duplicate *P. aeruginosa* isolates were collected from different clinical sources such as trachea (84/369), wound (51/369), urine (79/369), punch biopsy (62/369), blood (34/369), sputum (35/369) and other (24/369). These samples were obtained from patient hospitalized in intensive care (ICU) and neonatal ICU (174/369), internal (149/369), emergency (11/369), other (15/369) and 20 samples from outpatients referred to laboratory center [13].

A total of 29 non-duplicate *bla*_NDM-1_ positive *P. aeruginosa* were collected from different clinical samples. The identification of *P. aeruginosa* was done by the conventional microbiology tests and confirmed by PCR with specific primers for *gyrB* gene [14].

### PCR amplification of resistance genes

PCR amplification was performed for detection of *bla*_NDM_, *bla*_IMP_, *bla*_VIM_, *bla*_KPC_, *bla*_GES_, *bla*_SPM_ and *bla*_OXA-10_ using a set of specific primers on a thermal cycler (Eppendorf AG, Germany) as described previously [15–17]. Sequencing of the amplicons was performed by the Bioneer Company (Bioneer, Daejeon, South Korea) and the nucleotide sequences were analyzed using GenBank nucleotide database at http://www.ncbi.nlm.nih.gov/blast/.

### Antimicrobial susceptibility testing

Antibiotic susceptibility of the *bla*_NDM-1_ positive isolates was determined by the Kirby–Bauer method as recommended by the CLSI. The 11 standard antibiotic disks used include: imipenem (10 µg), meropenem (10 µg), ertapenem (10 µg), ceftazidime (30 µg), cefotaxime (30 µg), cefepime (30 µg), gentamicin (10 µg), piperacillin/tazobactam (100/10 µg), amikacin (30 µg), ciprofloxacin (5 µg) and aztreonam (30 µg) (Mast Group Ltd, UK). The ESBL phenotype was identified using combined disk method by disks of ceftazidime (30 mg) with (10 mg) and without clavulanic acid (Mast Group Ltd, UK), applied to all *bla*_NDM−1_ positive isolates (15). Moreover, the minimum inhibitory concentrations (MICs) of imipenem (10 µg/ml) [≤ 2 mg/L (susceptible), 4 mg/L (intermediate), and ≥ 8 mg/L (resistant)] (Liofilchem, Roseto degli Abruzzi, Italy) were applied by gradient test strips to *bla*_NDM−1_ positive *P. aeruginosa* isolates [18].

### Carbapenemase screening

The double-disk potentiation tests (DDPT) and double disk synergy test (DDST) was performed phenotypically for all *bla*_NDM−1_ positive described by Yong et al. [19].

### Double-locus sequence typing method

DLST typing was carried out using amplification of ms172 and ms217 loci using specific primers as previously described (Basset and Blanc, 2014) and according to DLST website (http://www.dlst.org/). PCR products were purified and were sequenced by Bioneer Corporation (Bioneer, Daejeon, South Korea).

**Fig. 1:**
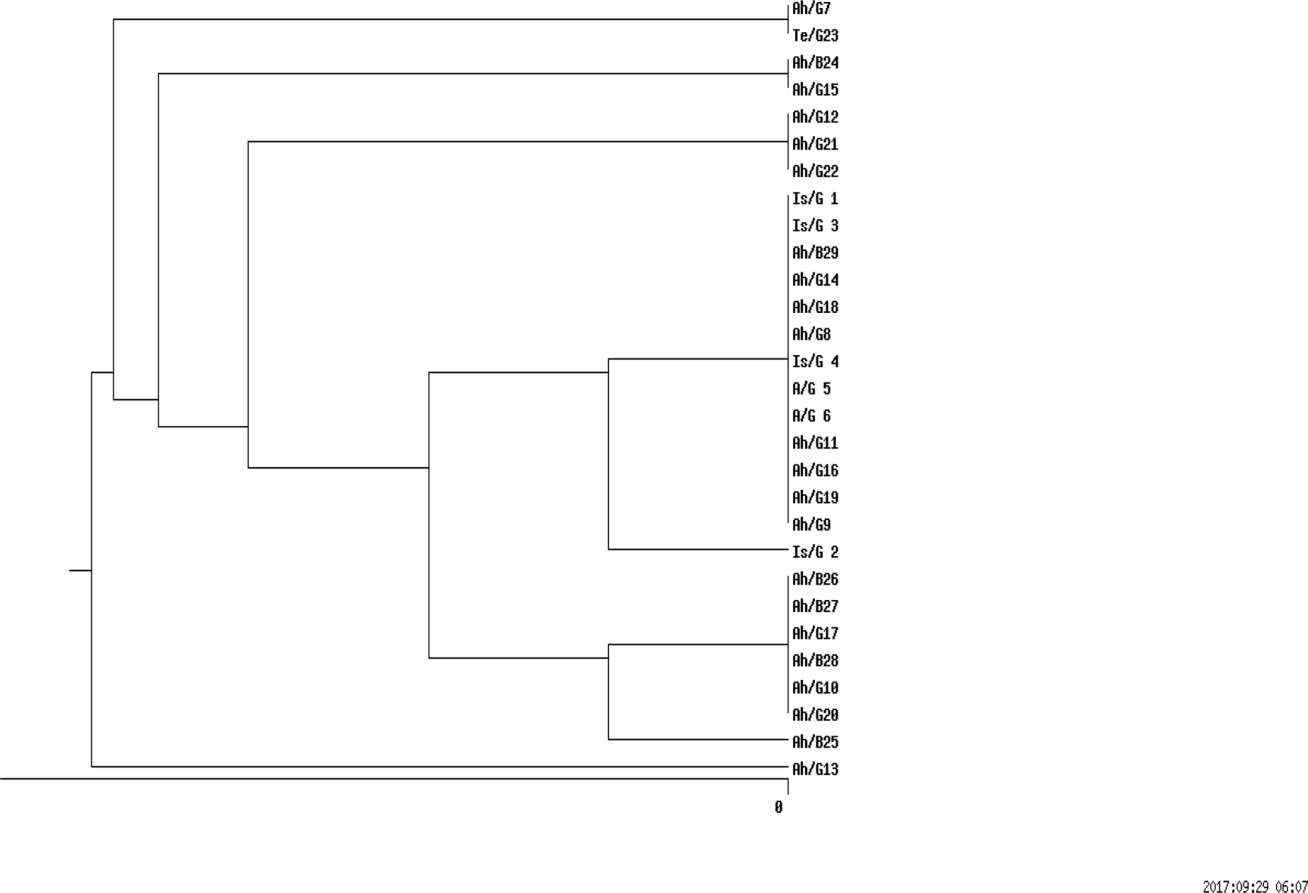
Dendrogram of DLST result analysis of 29 *bla*_NDM-1_ *P. aeruginosa* clinical strains isolated from hospitals in three different cities (Tehran, Ahvaz, Isfahan) of Iran. Results were clustered by UPGMA and compared by Dice Methods; Is; Isfahan, Ah; Ahvaz, Te; Tehran, G; General hospital, B; Burn hospital

In the cases of any results of allele assignment, the allele was considered as a null allele. The allele profiles were compared and clustered by the UPGMA and Dice methods (Fig. 1), using an online data analysis service (nslico.ehu.es).

## Results

During the three years of study, twenty-nine of *bla*_NDM-1_ positive isolates were collected, of them, 6 (20.7%) and 23 (79.3%) isolates were from burn patients and non-burn patients, respectively. The male to the female proportion in *bla*_NDM-1_ isolates was 3.22 (n= 20:9). The most *bla*_NDM-1_ positive strains were isolated from wound/punch (n = 11; 37.9%) followed by urine (n = 11; 37.9%) samples, whereas, the majority of the *bla*_NDM-1_ isolates were obtained from ICU ward (n = 21: 72.4%), followed by internal ward (n = 6; 20.7%) and burn ward (n = 6; 20.7%) (Table 1). Antibiotic susceptibility pattern showed that all isolates were resistance to imipenem and ertapenem, moreover, the resistance rate of meropenem was 96.5%. In contrast, the highest sensitivity was against to piperacillin/tazobactam and amikacin (41.4%). All *bla*_NDM-1_ positive isolates were defined as MDR. The full results of antibiotic resistance pattern of *bla*_NDM-1_ positive *P. aeruginosa* isolates showed in Table 2.

**Table 1:** The detailed results of *P. aeruginosa* with and without *bla*_NDM-1_

Variable	*P. aeruginosa *(without *bla*_NDM-1_) N=340 (%)	P. aeruginosa (with *bla*_NDM-1_) N=29 (%)	p-value
Sex			
Male	199 (58.5)	20 (69)	0.2
Female	141 (41.5)	9 (31)	
Type of sample			
Trachea	80 (23.5)	4 (13.8)	0.2
Urine	68 (20)	11 (37.9)	197
Punch	60 (17.6)	2 (6.9)	0.1
Wound	42 (12.3)	9 (31)	0.005
Sputum	34 (10)	1 (3.5)	0.2
Blood	32 (9.4)	2 (6.9)	0.6
Other	24 (70.5)	–	–
Type of patients			
Burn	45 (13.2295)	6 (20.7)	
ICU	153 (45)	21 (72.4)	0.09
Internal	143 (42.1)	6 (20.7)	0.02
Emergency	11 (3.2)	–	–
Outpatients	18 (5.3)	2 (6.9)	0.7
Other	15 (4.4)	–	–

*ICU* Intensive care unit

**Table 2:**
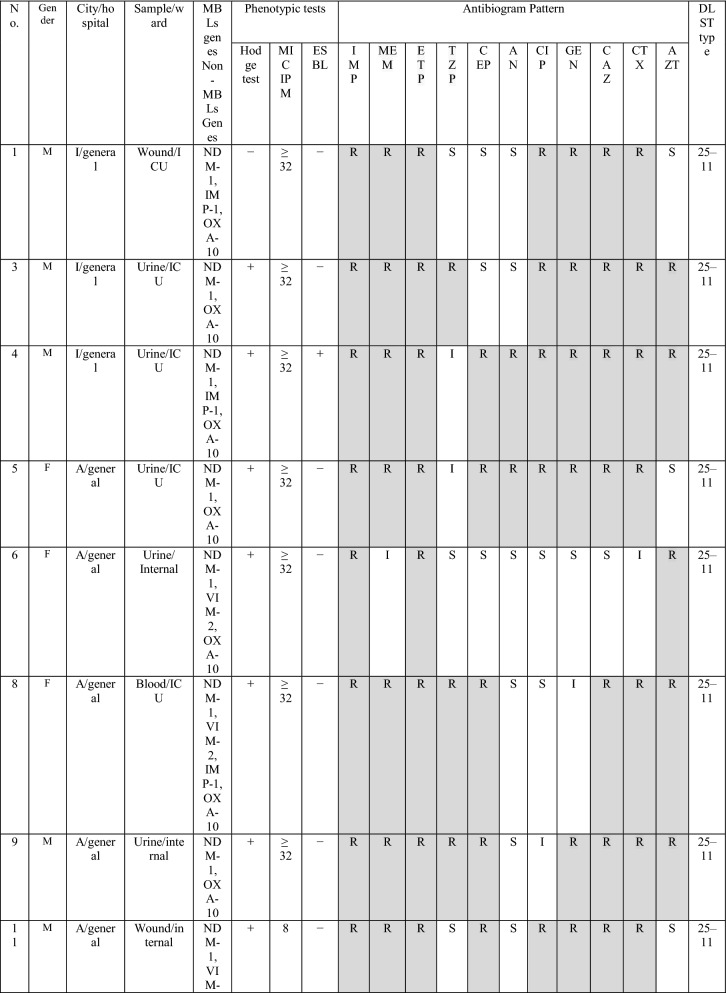

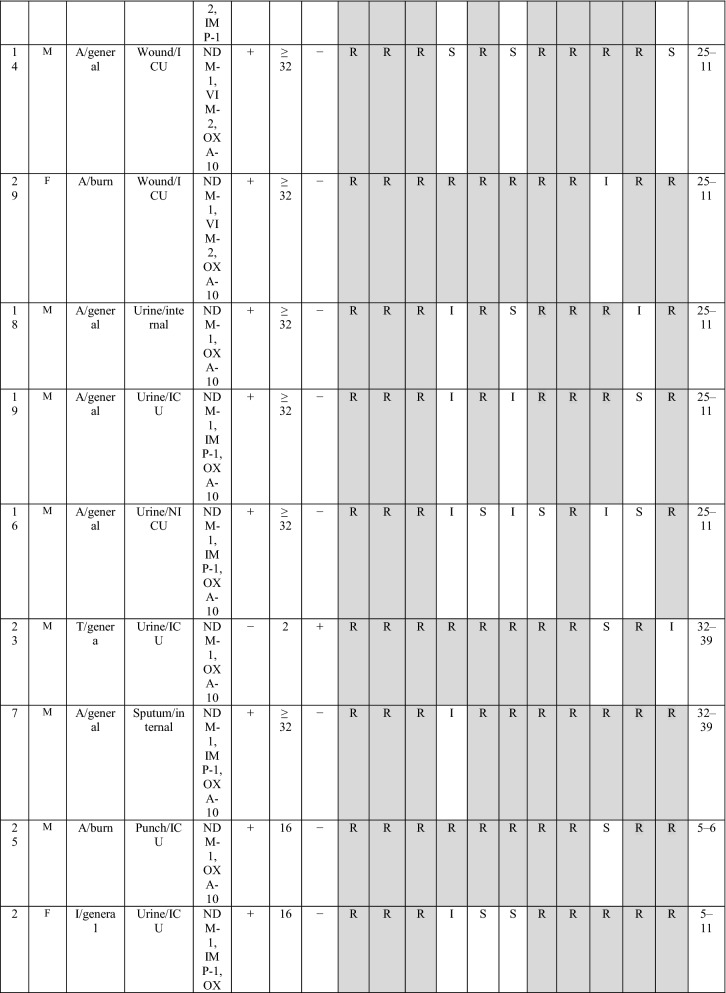

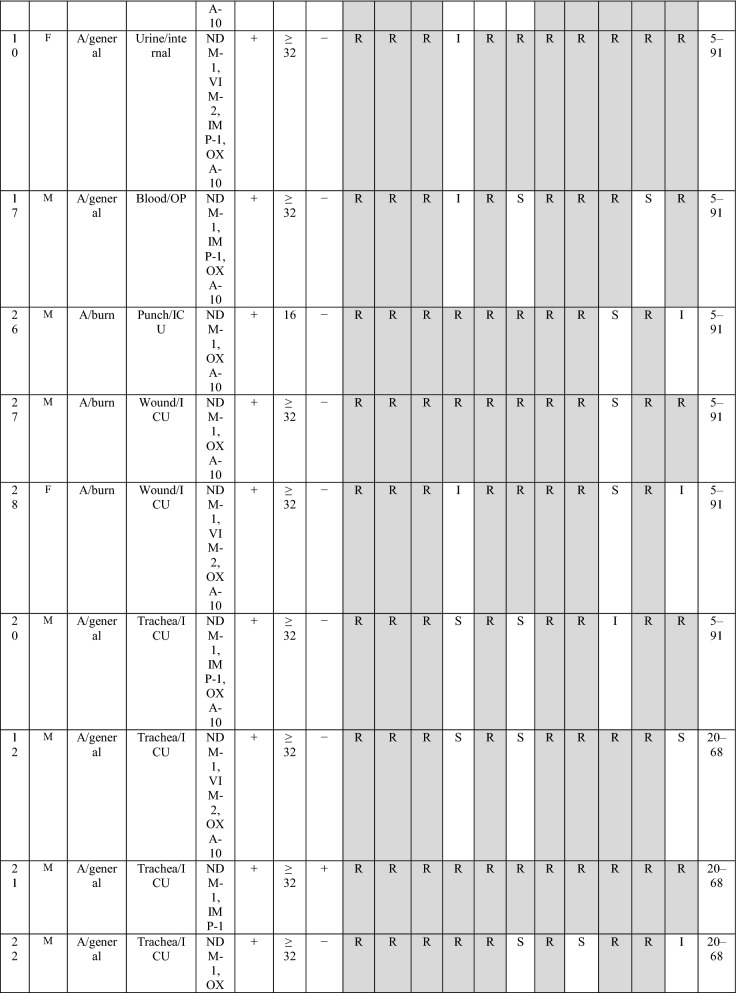

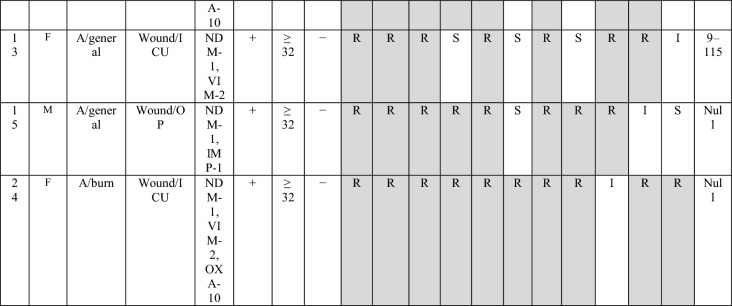
The detailed results of *bla*_NDM−1_ isolates

Imipenem (IPM), meropenem (MEM), ertapenem (ETP), piperacillin-tazobactam (TZP), cefepime (CFP), amikacin (AN), ciprofloxacin (CIP), gentamicin (GEN), ceftazidime (CAZ), cefexime (CTX), azteronam (AZT).َ A, Ahvaz; I, Isfahan; T, Tehran; OP, Outpatient; ICU

The MICs of imipenem against *bla*_NDM−1_ positive *P. aeruginosa* isolates are presented in Table 1. Overall, the results of MICs of imipenem showed 82.7% (24/29) isolates were high-level imipenem-resistant isolates (MIC ≥32). DDST and DDPT revealed that 15/29 (51.8%) and 26 (89.7%) of *bla*_NDM-1_ positive isolates were MBL producing isolates, respectively. In addition, the results of MHT showed that 27/29 (89.6%) of *bla*_NDM−1_ positive *P. aeruginosa* isolates were carbapenemase-producing isolates.

The distribution of carbapenemase genes and other antibiotic resistance genes among *bla*_NDM-1_ positive *P. aeruginosa* isolates are presented in Table 2. However, PCR analysis showed none of the *bla*_NDM-1_ positive *P. aeruginosa* isolates contained *bla*_KPC_ and *bla*_GES_ genes. The presence of *bla*_VIM-2_, *bla*_IMP-1_ and *bla*_SPM_ genes were detected in 41.4%, 34.5% and 3.5% isolates, respectively. Among *bla*_NDM-1_ positive *P. aeruginosa* isolates, the *bla*_OXA-10_ beta-lactamase was the most frequently gene recognized in 86.2% (25/29).

The combination of *bla*_VIM-2/_ *bla*_OXA-10_ and *bla*_IMP-1/_*bla*_OXA-10_ beta-lactamase was found in 8 (27.6%) and 10 (34.5%) isolates, respectively. Moreover, among *bla*_NDM-1_ positive *P. aeruginosa* isolates the co-harboring of three genes, *bla*_OXA-10_, *bla*_IMP-1_ and *bla*_VIM-2_ was found in two isolates and only one isolate contained *bla*_SPM_ that in combination with *bla*_VIM-2_ and *bla*_OXA-10_.

In the current study, the DLST method was tested for *all bla*_NDM-1_ isolates recovered over a period of four years from various hospital wards. DLST results revealed partial diversity among 29 *bla*_NDM-1_ positive isolates. Totally, 8 different DLST profile (DL type) (four different common types and three single type) were detected (Table 2). The most common type including 13 isolates (45%) from different hospitals (in Ahvaz and Isfahan). A total of 29, 27 sequences were ms172 and ms217 whereas, 2 strains carried null alleles for these loci. The DLST profile 5–91 were detected in 3 (50%) burn isolates. The details of information about DL type of *P. aeruginosa* isolates are presented in Table 2.

## Discussion

*P. aeruginosa*, one of the most common opportunistic pathogen associated with nosocomial infections, including pneumonia, urinary tract infections, and wound infections [1, 20]. Although carbapenems are often used as a therapeutic agent for treating infections caused by *P. aeruginosa*, the high emergence of carbapenem resistance significantly decreases their usefulness [21, 22]. The presence of *bla*_NDM−1_ producing isolates which may increase resistance to carbapenems is increasing among patients in healthcare systems [23]. In the current study, we described molecular characterization of *bla*_NDM-1_ producing of *P.aeruginosa* isolates with phenotypic and genotypic methods.

The overall data show that the frequency of *bla*_NDM-1_ producing *P. aeruginosa* isolates was 7.8% (29/369). In addition, there are variable reports of *bla*_NDM-1_ from different countries in Europe and Asian. The *bla*_NDM-1_ producing *P. aeruginosa* isolates has been also detected in Iran, recently. Shokri et al. from Isfahan (Center of Iran) in 2017 reported 6% of *P. aeruginosa* isolates were *bla*_NDM-1_ positive which is slightly lower than the result obtained in the present study [24]. In another study, Dogonchi et al. reported one isolate of *P. aeruginosa* harboring *bla*_NDM-1_ in the north of Iran [25]. Recently, Azimi et al. described a lower frequency rate for *bla*_NDM-1_ (7%) among carbapenem-resistant *P. aeruginosa *[26]. Moreover, the *bla*_NDM−1_ gene was also revealed by Riahi Rad et al. in Iran (21.4%) [27] and Takahashi et al. in Nepal (7%) [28].

With regard to the fact that our isolates were collected from hospitals of different cities in Iran and also regarding the results of previous studies, an increasing trend of *bla*_NDM-1_ producing *P. aeruginosa* strains can be observed in Iranian hospitals where it could be an endemic and serious concern in future. One of the important reason of possible increase of this phenomenon among Gram-negative isolates is inappropriate and excessive prescription and use of carbapenems in our hospitals, which leads to selective pressure.

According to our results, the most *bla*_NDM-1_ positive isolates (72.4%) were collected from the ICU ward that these findings are broadly consistent with the previous studies conducted in Iran [24, 25]. This finding suggests that the ICU ward is can be a risk factor and major source for the dissemination of resistant genes in the Iranian hospitals. Our results presented that *bla*_NDM-1_ positive isolates had highly resistant to all antibiotics commonly used in the clinic which is in agreement with to the results of other studies [24, 29, 30].

In spite of the fact that the *bla*_NDM-1_ gene demonstrating the sensitivity of bacteria to aztreonam, 62% of *bla*_NDM-1_ positive isolates were resistant to this agent that could be related to the presence of other beta-lactamase genes. Based on screening of other carbapenemase and metallo β-lactamase genes, *bla*_OXA-10_ was the most frequently detected beta-lactamase among *bla*_NDM-1_ positive strain and *bla*_IMP-1_ was second, which is in contrast to other reports where *bla*_VIM_ was significantly associated with *bla*_NDM-1_ [31, 32].

One of the important findings in this study was the emergence of the co-harboring of *bla*_NDM-1_ positive *P. aeruginosa* isolates with more than one carbapenemase gene and metallo β-lactamase determinants, simultaneously. Accordingly, we report the first isolate of *P. aeruginosa* producing four carbapenemases co-existence *bla*_NDM-1,_ *bla*_VIM--2_, *bla*_IMP-1_ and *bla*_OXA-10_ from Iran. One of them that was obtained from urine sample was resistant to all antibiotics used except to TZP that were intermediate. Furthermore, we demonstrated the co-harboring of *bla*_NDM-1_ with metallo-β-lactamases genes such as *bla*_OXA-10_, *bla*_IMP-1_ and *bla*_VIM-2_ in *P. aeruginosa.* The coexistence of carbapenemases encoding genes with *bla*_NDM-1_ positive *P. aeruginosa* isolates has been reported in several Asian and European countries including in India (*bla*_NDM-1_ + *bla*_IMP_+ *bla*_VIM_+ *bla*_SPM)_, Denmark (*bla*_NDM-1_ + *bla*_VIM-5_+ *bla*_VIM-6_) [33], Bangladesh (*bla*_NDM-1_, *bla*_VIM-1_, *bla*_VIM-2_, *bla*_IMP-1_) [34] and Turkey (*bla*_VIM-1_+ *bla*_VIM-2_+ *bla*_GES-5_ [35].

The previous studies revealed that the acquisition of MBL determinants such as *bla*_NDM-1_, *bla*_VIM_, *bla* IMP and *bla*_SPM_ led to the emergence of MDR or XDR *P. aeruginosa* [36, 37].

To the extent of our knowledge, this study is the first to report of Molecular typing of *bla*_NDM-1_ positive isolates in *P. aeruginosa* by DLST method.

Among various genotyping technique, DLST as a reliable genotyping method, provide a new, rapid, typability, stability and low-cost of epidemiological surveillance of *P. aeruginosa* isolates [11, 38]. Analysis of DLST types revealed that the majority (19/29) of the isolates belonged to DLST type 25-11 and 5-91.

In addition, 8 *bla*_NDM-1_ positive isolates were clustered into two DLST types and 3 singletons. Accordingly, *bla*_NDM-1_ positive isolates were relatively heterogeneous, however, the route of transmission is not clear. These results highlight the importance of investigating carbapenem-resistant *P. aeruginosa* isolates in health care settings in our region.

## Conclusions

The occurrence of *bla*_NDM-1_ isolates in *P. aeruginosa* is a large challenge in the treatment and worrying for global health. DLST type 25-11 is a significant cluster because a large number of *bla*_NDM-1_ isolates showed this genotype and also DLST type 5-91 known as an alarming type in burn patients. This work suggests that the DLST as an appreciated method in typing of *bla*_NDM−1_ strains; this technique reducing considerably the time and the cost of the molecular analysis and providing a reliable phylogenetic study. This information can help to generate the proper strategies for accurate and specific use of this antibacterial which can help to control of *bla*_NDM-1_ isolates.

## Data Availability

Not applicable.

## Abbreviations

ESBL: Extended-spectrum b-lactamases
CLSI: Clinical and Laboratory Standards Institute
CCs: Clonal complex
DLV: Double-locus variants
DDST: Double-disk synergy test
DDPT: Double-disk potentiation test
KTP: Kidney transplant patients
ST: Sequence types
SLV: Single-locus variants
SD: Standard deviation
UPEC: Uropathogenic *Escherichia coli*
UTI: Urinary tract infections
MLST: Multilocus sequence typing
VFs: Virulence factors
