# Correction to: Molecular characterization of *NDM-1*-producing *Pseudomonas aeruginosa* isolates from hospitalized patients in Iran

**DOI:** 10.1186/s12941-021-00484-1

**Published:** 2021-12-11

**Authors:** Mojtaba Shahin, Ali Ahmadi

**Affiliations:** 1 Molecular Biology Research Center, Systems Biology and Poisonings Institute, Baqiyatallah University of Medical Sciences, Tehran, Iran; 2 Department of Medical Laboratory Sciences, Faculty of Medical Sciences, Arak Branch, Islamic Azad University, Arak, Iran

## Correction to: Ann Clin Microbiol Antimicrob (2021) 20:76 https://doi.org/10.1186/s12941-021-00482-3

Following publication of the original article [1], the affiliation details for the corresponding author “Ali Ahmadi” were incorrectly given as “Department of Medical Laboratory Sciences, Faculty of Medical Sciences, Arak Branch, Islamic Azad University, Arak, Iran” but should have been “Molecular Biology Research Center, Systems Biology and Poisonings Institute, Baqiyatallah University of Medical Sciences, Tehran, Iran”. These have been corrected with this erratum.

The original article has been corrected.
